# Supplementary material for: Application of Machine Learning Methods to Investigate Joint Load in Agility on the Football Field: Creating the Model, Part I
Source: Sensors (Basel). 2024 Jun 5;24(11):3652. doi: 10.3390/s24113652 (PMC11175175; doi:10.3390/s24113652)
Supplement: Supplementary file 1 [file sensors-24-03652-s001.zip › sensors-2995499-supplementary.pdf]

Classification models	Regression Models
**Decision Trees**	**Regression Trees**
Fine Tree	Fine Tree
Medium Tree	Medium Tree
Coarse Tree	Coarse Tree
**Discriminant Analysis**	**Linear Regression Models**
Linear Discriminant	Linear
Quadratic Discriminant	Interactions Linear
**Logistic Regression**	Robust Linear
Binary GLM Logistic	Stepwise Linear
**Naive Bayes**	**Support Vector Machines (SVM)**
Gaussian Naive Bayes	Linear SVM
Kernel Naive Bayes	Quadratic SVM
**Support Vector Machines (SVM)**	Cubic SVM
Linear SVM	Fine Gaussian SVM
Quadratic SVM	Medium Gaussian SVM
Cubic SVM	Coarse Gaussian SVM
Fine Gaussian SVM	**Ensemble Methods**
Medium Gaussian SVM	Boosted Trees
Coarse Gaussian SVM	Bagged Trees
**k-Nearest Neighbors (k-NN)**	**Gaussian Process Regression**
Fine kNN	Rational Quadratic
Medium kNN	Squared Exponential
Coarse kNN	Matern 5/2
Cosine kNN	Exponential
Cubic kNN	**Neural Networks**
Weighted kNN	Narrow Neural Network
**Kernel Approximation Methods**	Medium Neural Network
SVM Kernel	Wide Neural Network
Logistic Regression Kernel	Bilayer Neural Network
**Ensemble Methods**	Trilayer Neural Network
Boosted Trees	**Kernel Approximation Regression Methods**
Bagged Trees	SVM Kernel
RUS Boosted Trees	Least Squares Regression Kernel
Subspace Discriminant	
Subspace kNN	
**Neural Networks**	
Narrow Neural Network	
Medium Neural Network	
Wide Neural Network	
Bilayer Neural Network	
Trilayer Neural Network	

Table S1. Full list of machine learning models adopted in the study separately for classification and regression problem. The analyses were run in Classification Learner and Regression Learner of the Matlab *Statistics and Machine Learning Toolbox* (vR2022a).
